# NAD^+^-A Hub of Energy Metabolism in Heart Failure

**DOI:** 10.7150/ijms.89370

**Published:** 2024-01-01

**Authors:** Yaoxin Wu, Zuowei Pei, Peng Qu

**Affiliations:** 1 Faculty of Medicine, Dalian University of Technology, 116024, Dalian, China.; 2 Department of Cardiology, Central Hospital of Dalian University of Technology, Dalian, 116033, China.

**Keywords:** NAD^+^, Energy Metabolism, Heart Failure, Mitochondria

## Abstract

Heart failure is a condition where reduced levels of adenosine triphosphate (ATP) affect energy supply in myocardial cells. Nicotinamide adenine dinucleotide (NAD^+^) plays a crucial role as a coenzyme for electron transfer in energy metabolism. Decreased NAD^+^ levels in myocardial cells lead to inadequate ATP production and increased susceptibility to heart failure. Researchers are exploring ways to increase NAD^+^ levels to alleviate heart failure. Targets such as sirtuin2 (sirt2), sirtuin3 (sirt3), Poly (ADP-ribose) polymerase (PARP), and diastolic regulatory proteins are being investigated. NAD^+^ supplementation has shown promise, even in heart failure with preserved ejection fraction (HFpEF). By focusing on NAD^+^ as a central component of energy metabolism, it is possible to improve myocardial activity, heart function, and address energy deficiency in heart failure.

## 1. The Energy Metabolism of Myocardial Cells and Heart Failure

### 1.1. Energy Metabolism in the Heart

The heart is one of the important metabolic organs in the human body, which promotes the blood circulation of the whole body through its relaxation and contraction. Like other cells, the source of energy for healthy human myocardial cells is ATP. However, for myocardial cells, fatty acids are the primary substrate for ATP production in myocardial cells, contributing 50%-70% of the ATP demands 1, other substrates such as lactate, ketone bodies, glucose, and branched-chain amino acids (BCAAs) are utilized by myocardial cells for ATP production as well 2. Glucose is also an important energy material for the heart. In myocardial cells, a small amount of ATP and pyruvic acid can be produced by cytoplasmic glycolysis, and pyruvic acid enters the mitochondria to produce more ATP. Under the condition of increased circulating lactic acid levels, lactic acid could also be used as an important energy substrate for the heart. The heart takes up lactic acid through the monocarboxylic acid anion transporter (MCT4) and converts it into pyruvic acid using lactate dehydrogenase (LDH); the pyruvic acid is then utilized in mitochondrial metabolism to produce ATP 3, 4. Ketone bodies are easily metabolized by the heart. When the level of ketone bodies increases in the circulation, the heart switches to using ketone bodies as its primary fuel source 5, this process is achieved by regulating the phosphorylation level in the heart and the concentration of mitochondrial NADH 6. A growing body of research supports the notion that ketone bodies play a significant role as energy substrates for the heart 7, 8. Amino acid oxidation is considered a significant potential source of ATP production in myocardial cells, with branched-chain amino acids (BCAAs) being particularly optimal in this regard 9. Therefore, in terms of energy metabolism, the heart has extremely high flexibility, which can flexibly select among different substrates under different conditions to ensure adequate ATP supply, thereby providing sufficient conditions for the heart to complete its functions.

### 1.2. Energy Metabolism of Myocardial Cells During Heart Failure

Heart failure (HF) refers to a disease of heart circulation disorder caused by heart systolic and/or diastolic dysfunction, resulting in blood stasis in the venous system and insufficient blood perfusion in the arterial system and massive venous return to the heart. It is generally divided into heart failure with reduced ejection fraction (HFrPH), HFpEF and heart failure with mid-range ejection fraction (HFmrEF) according to left ventricular ejection fraction 10. HFpEF represents approximately 50% of all cases of heart failure, and its morbidity and mortality rates are comparable to those of heart failure with reduced ejection fraction, but the underlying mechanisms and pathogenesis of HFpEF are not yet well understood 11, 12. For heart failure, on the one hand, it loses metabolic activity, and on the other hand, it becomes energy deficient due to the decreased ability to produce ATP. Existing studies have shown that the above energy deficiency may be due to the decreased mitochondrial oxidation ability in heart failure. The common causes of mitochondrial injury in heart failure include the following: (1) Increased production of reactive oxygen species (ROS) and disruption of mitochondrial calcium (Ca^2+^) homeostasis; (2) Mitochondrial kinetic injury, persistent mitosis, autophagy of myocardial cells, and elevated cell death; (3) Alterations in the transcriptional regulation of mitochondrial proteins and increased post-translational protein modifications 2. Although various abnormalities related to calcium homeostasis, contractile protein function, energy metabolism, cytoskeletal rearrangements, and loss of viable muscle cells have been observed, the fundamental molecular and cellular mechanisms underlying the pathogenesis of heart failure are not yet well understood 13. It should also be noted that, as mentioned above, myocardial cells have great flexibility in selecting substrates for energy metabolism. Studies have shown that in the early stage of HF, the selection of myocardial substrates is relatively normal; However, in the late stage, fatty acid oxidation is down-regulated, glycolysis and glucose oxidation are increased, respiratory chain activity is reduced, and mitochondrial oxidation flux reserve is damaged 14, which are the reasons for insufficient myocardial energy supply during HF.

### 1.3. NAD(H) of Energy Metabolism in Heart Failure

Intracellular NAD exists in both oxidized form (NAD^+^) and reduced form (NADH), and it serves as the primary electron carrier coenzyme during energy metabolism processes such as glycolysis, oxidative phosphorylation, and the tricarboxylic acid cycle (TCA) 15. Studies have demonstrated that reductions in NAD or NAD^+^/NADH levels are observed in heart failure 16, 17. Additionally, relevant studies have confirmed the beneficial effects of providing NAD^+^ precursors in clinical patients with heart failure as well as in preclinical models 18. Numerous studies indicate that the metabolic level of NAD^+^ in heart failure is associated with sirtuins, PARP (poly (ADP-ribose) polymerase), and cyclic ADP-ribose synthase (CD38) 19, 20. These enzymes use NAD^+^ as metabolic substrate in heart failure. Increasing the level of NAD^+^ has emerged as a new research direction for alleviating and treating heart failure. Two key issues in this area include: (1) understanding the impact of low heart NAD^+^ levels on the pathogenesis of heart failure, and (2) elucidating the mechanisms by which elevated NAD^+^ levels provide benefits in heart failure treatment.

#### 1.3.1. Research Status of NAD^+^

Increasing NAD^+^ levels is considered a promising strategy for treating heart failure, although the mechanistic details may vary. Various compounds, including NAD^+^ precursors or nicotinamide phosphotransferase (Nampt) activators, have shown potential in this regard 21. However, there is currently a lack of information regarding the pharmacokinetics and drug tolerance of these compounds in patients. Diguet et al. developed and published the dynamics of NAD^+^ metabolomics in human subjects after administering a single dose of Nicotinamide ribonucleoside (NR) 22. Another study investigated the pharmacokinetics of NR and its impact on blood NAD^+^ levels in healthy volunteers over a nine-day treatment period 23. The above research undoubtedly made great progress in the treatment of heart failure with NAD^+^, but the current research has always had limitations. One point is that the current detection method is to measure the levels of NAD^+^ in blood, and the changes of NAD^+^ levels in heart and other metabolic conditions cannot be determined by NAD^+^ levels in blood alone. While many preclinical studies have taken a precautionary approach by inducing heart failure and expanding the NAD^+^ pool simultaneously, for clinical applications, it is more appropriate to treat individuals who already have heart dysfunction 21. The focus shifts to administering NAD^+^-boosting therapies to subjects with existing heart dysfunction for potential clinical benefits.

## 2. The Relationship Between NAD^+^ and Heart Failure

### 2.1. Role of NAD^+^ Consumption and Synthesis Pathway in the Heart

It is well known that the intracellular NAD pool consists of two forms, the oxidized form (NAD^+^) and the reduced form (NADH), which will carry electrons from substrate decomposition for oxidative phosphorylation and biosynthesis. Intracellular NAD^+^ serves as a synergistic substrate for PARPs, sirtuins, and CD38, and during these enzymatic reactions, nicotinamide (NAM) is produced 24. PARP (poly (ADP-ribose) polymerase) is a multifunctional DNA-binding enzyme found in various nuclei, including myocardial cells 25. It can be activated by a single-strand DNA cleavage reaction, which may result from oxidative cell injury and the presence of free radicals. Mild activation of PARP (poly (ADP-ribose) polymerase) under physiological conditions regulates several cellular processes including DNA repair, gene expression, cell cycle progression, cell survival, chromatin remodeling, and maintenance of genomic stability 26. However, excessive activation of PARP (poly (ADP-ribose) polymerase) leads to the depletion of cellular NAD^+^ content 7. This, in turn, inhibits other NAD^+^-dependent cellular processes. Sirtuins are a crucial class of deacetylating proteins involved in numerous physiological processes. There are several types of sirtuins, including sirtuin 1-7 (sirt1-7), each playing distinct roles in cellular regulation 27. Endogenous sirtuin 1 (sirt1) plays a critical role in mediating cell death an 28. Sirtuin 3 (Sirt3) protects myocardial cells from aging, oxidative stress, and inhibits heart hypertrophy 29. Sirt1 regulates mitochondrial function through acetylated nuclear proteins, while sirt3 regulates mitochondrial function through acetylated mitochondrial proteins 30. Eukaryotes typically synthesize NAD^+^ through the ab initio pathway using tryptophan or salvage it from NAM. In the salvage pathway, NAM is converted to nicotinamide mononucleotide (NMN) by the enzyme Nampt. Additionally, phosphorylation of nicotinamide ribonucleoside kinase (Nrk) can generate NMN from NR 31.

### 2.2 Heart Changes Caused by Reduced NAD^+^ Levels

In cell metabolism, the activities of acyl-coenzyme-A dehydrogenase and 3-hydroxyacyl coenzyme-A dehydrogenase are influenced by flavin adenine dinucleotide (FAD) / reduced flavin adenine dinucleotide (FADH2) and the ratio of NAD^+^/NADH, respectively. These factors, in turn, impact oxidative phosphorylation and ATP synthesis. Based on existing research, both human and HFpEF rat studies have shown preserved expression of Nampt in the heart 2. This suggests that the decrease in NAD^+^ content may be due to increased NAD^+^ consumption or a low level of substrate nicotinamide available for Nampt. In SRFHKO mice with heart failure, the NMRk2 pathway is activated. This activation leads to an increase in the expression of NT5E, which is the extracellular enzyme responsible for hydrolyzing extracellular NAD^+^ and NMN to Nicotinamide nucleoside (NR) 27. In addition, in the tissue biopsy of heart failure, since the increase of active oxygen and oxygen free radicals, the expression and activity of PARP1 are increased. On the one hand, PARP1 can degrade its substrate NAD^+^ leading to a reduction in NAD^+^ levels. On the other hand, PARP1 inhibits the phosphoinositide 3 (PI3)-kinase-Akt signaling pathway, which hampers Akt's ability to inhibit muscle cell death-promoting enzymes such as c-Jun N-terminal kinase (JNK) and P38. Conversely, inhibiting PARP-1 can enhance Akt activation, thereby mitigating heart injury, reducing fibrosis, and protecting heart function 32. Studies have found that the reduced level of NAD^+^ in cell is actually the cause of PARP-mediated cardiomyocyte death. Reduced activity of the sirtuin isoform, sirt2 deacetylase, contributes to PARP-mediated myocyte death. However, activation of this enzyme can prevent PARP-mediated NAD^+^ depletion and subsequent cell injury 33.

### 2.3 Improvement of Heart Failure and its Mechanisms by Increasing NAD^+^ Levels

As mentioned above, ATP is in short supply in heart failure, and the key reason for ATP deficiency is the low level of NAD^+^. Therefore, supplementing and increasing NAD^+^ level has become a feasible scheme to treat heart failure and alleviate heart injury. As a direct precursor in the synthesis of NAD^+^, NMN plays a direct role in improving the level of NAD^+^. A study using KFL4 deficient mice (with decreased NAD^+^ level) shows that NMN can protect the ultrastructure of mitochondria, reduce reactive oxygen species, prevent the death of heart cells, and increase the oxidation of long-chain fatty acids, thereby increasing the NAD^+^ level 34. Another experiment using p32cKO mice as a heart failure model shows that NMN can also alleviate heart failure by improving the function of lysosomes and reducing autophagy 35. NMN has the same effect on HFpEF. Studies have shown that oral supplementation of its precursor nicotinamide to increase NAD^+^ can improve diastolic dysfunction induced by aging (in 2-year-old C57BL/6J mice), hypertension (in Dahl salt-sensitive rats) or cardiac metabolic syndrome (in ZSF1 obese rats) 36. Another study on Nampt shows that as a rate-limiting enzyme for synthesizing NAD^+^ from NMN, its activity is very important for the remediation of NAD^+^ through NMN 37. The latest clinical study on NMN can directly show that NMN can increase the level of NAD^+^ and help restore energy metabolism 38. Another clinical study also shows that oral NMN is a safe and practical strategy to improve the level of NAD^+^ in human body 39. Due to the reaction that NR is phosphorylated by Nrk to produce NMN, NR supplementation has been mainly used in the existing studies 40. NR increases the levels of heart nicotinic acid adenine dinucleotide, a sensitive biomarker of increased NAD^+^ metabolism, and methyl NAM (MeNAM), which is oxidized by aldehyde oxidase (AOX1) to N1- methyl -4- pyridone -5- carboxamide (Me4PY) and releases hydrogen peroxide 41. NR has the potential to improve both congenital and acquired heart failure 42. Furthermore, NR may also have systemic effects, particularly in liver metabolism, insulin antagonism, and skeletal muscle function, which can be altered in conjunction with heart defects 43. When NR treatment is performed, the induction level of Myh7 in SRFHKO mice is limited, and Myh7 induction is a signal of heart stress and metabolic remodeling in heart failure 44. NR also increases mitochondrial citrate synthase (CS) and TP- citrate lyase (ACL) activity, leading to increased nucleoprotein acetylation (Acetyl coenzyme A precursor
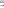
citrate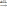 Acetyl coenzyme A) 45. Due to the reduced concentration of NAD^+^ in muscle cells, PARP1 can attenuate the activity of sirts deacetylase in muscle cells. On the contrary, overexpression of sirt1 by increasing the level of NAD^+^ prevents PARP-1-induced depletion of NAD^+^, while the deacetylation of sirts inhibits the effects of P53 and Ku70 46. In studies conducted on the heart, analysis of acetylated proteins revealed that supplementation with nicotinamide resulted in the deacetylation of two diastolic regulatory proteins, titin and SERCA2a 47. SERCA2a is a direct substrate for sirt1 and P300 (histone acetyltransferase P300). sirt1 is activated by the metabolic activator β-lapachone (β-lap) of sirt1, which significantly reduces acetylation and recovers the function of SERCEAA, thereby alleviating heart failure. In addition, as mentioned above, ROS are increased in heart failure, while supplementation of NAD^+^ by NR has been shown to reduce mtROS of different tissue types 48-52.

Although there are few studies on HFpEF at present, it is still feasible to treat HFpEF by increasing NAD^+^. On the one hand, NR has the ability to directly enhance the passive stiffness of myocardial cells and improve active calcium-dependent relaxation. This is achieved by increasing the deacetylation of titin, which impacts passive stiffness, and the sarcoplasmic reticulum calcium-ATPase 2a (SERCA2a) enzyme, which influences active relaxation 53. On the other hand, sirt3 is related to ATP synthase under normal mitochondrial membrane potential, but it is released to activate the TCA when the mitochondrial membrane potential is reduced. However, the positive feedback loop needs sirt3 to activate NMNAT3 54. Therefore, increasing the NAD^+^ level can increase sirt3 activity and alleviate the hyperacetylation of the related mitochondrial enzymes. Studies have found that the increased acetylation of very-long chain acyl-CoA dehydrogenase deficiency (VLCAD) and hydratase subunit A (HADHA) can decrease the fatty acid oxidation (FAO) of HFpEF-derived myocardial cells, and the supplementation of NAD^+^ can effectively reduce the FAO defect 10. In another study on HFpEF, it was found that NR supplementation can normalize NAD^+^/NADH ratio, down-regulate acetylation level, improve mitochondrial function and improve HFpEF phenotype 55. In addition to NR, NAM can also increase the level of NAD^+^. It has been found that NAM can improve the systemic HFpEF risks of ZSF1 obese rats, including obesity and hypertension. Similarly, NAM may also improve diastolic function through the deacetylation of titin and SERCA2a. Titin improve the passive stiffness of myocardial cells while SERCA 2a improve the dependent active relaxation 36.

In addition to promoting the synthesis of NAD^+^ by increasing the precursor of NAD^+^, the existing research shows that this goal can be achieved by directly using NAD^+^. In a study on the regulation of NAD^+^ on neuroinflammation, the level of NAD^+^ can also be increased by direct intraperitoneal injection of NAD^+^ instead of its precursor 56. In another study on the mouse model with high-fat diet, the researchers also adopted the experimental method of intravenous injection. The experimental results showed that intravenous injection of NAD^+^ could save the cells in hypothalamic neurons from NAD^+^ depletion and its induced inhibition of PER1 transcription activity 57. Another study on the energy supply system of left and right ventricular myocardium after using intravenous injection of NAD^+^ to restore short-term circulatory disorder shows that intravenous injection of NAD^+^ is helpful to oxidative phosphorylation of myocardial cells 58. However, it should be noted that the structure of NAD^+^ itself is unstable and it cannot be taken orally, so the direct injection of NAD^+^ is still under study, especially about HF.

## 3. Conclusion and Prospect

Although the mechanism of changes about NAD(H) levels in heart failure is not fully understood, the action targets for increasing NAD^+^ levels to treat heart failure are not clear. On the basis of existing studies, such as involving relevant mechanisms as sirts, PARPs, troponin, and contraction/relaxation protein, as well as supplementing information on pharmacokinetics and drug toxicology, NAD^+^ as a therapeutic direction for heart failure will further prove to be completely feasible. In conclusion, the NAD^+^ therapy is an effective idea for treating heart failure and alleviating heart injury. It is believed that with further research and further supplement of relevant information, the mechanism and efficacy under relevant scenarios will be better understood.

## Figures and Tables

**Figure 1:**
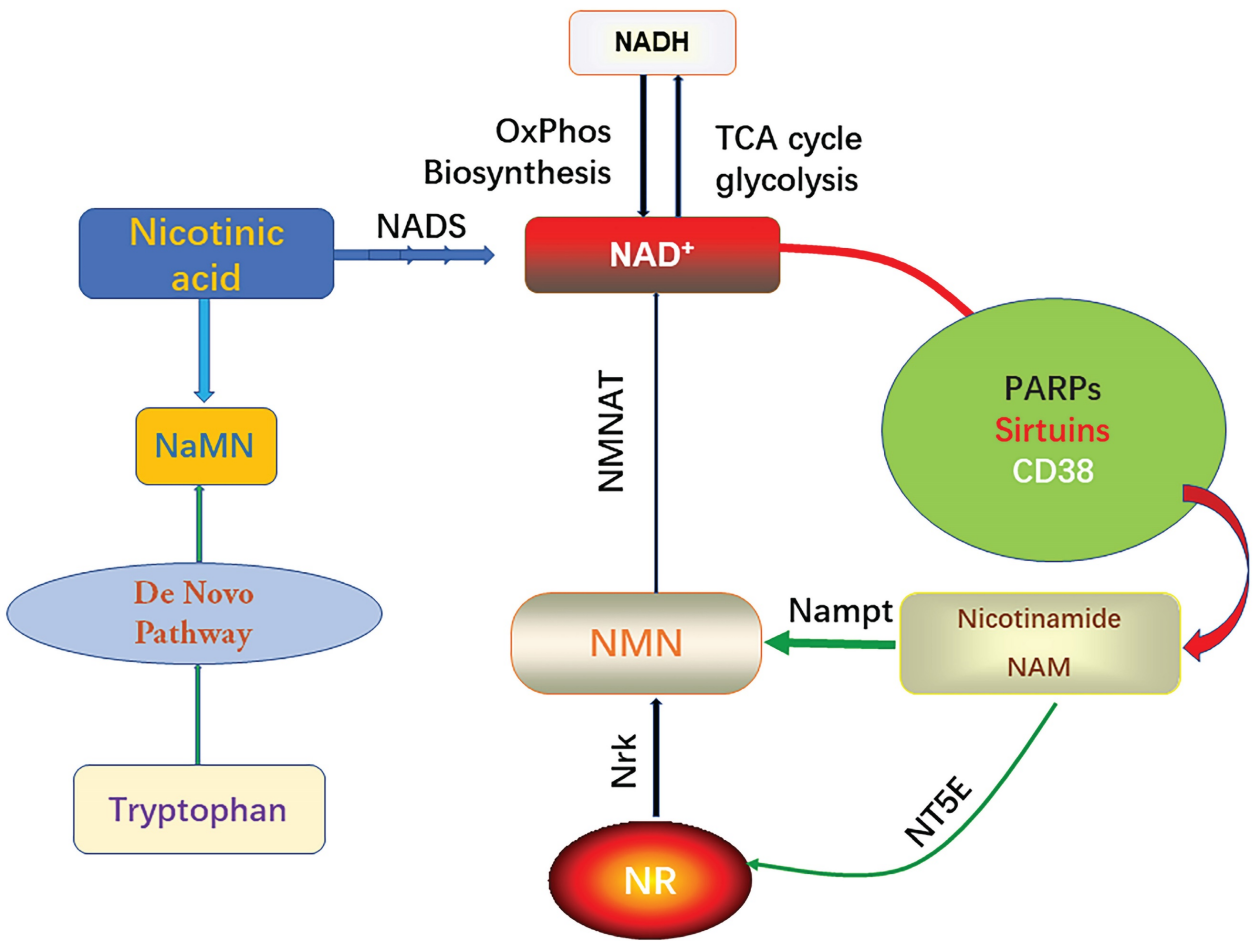
Biosynthesis and consumption pathway of NAD^+^.

**Figure 2:**
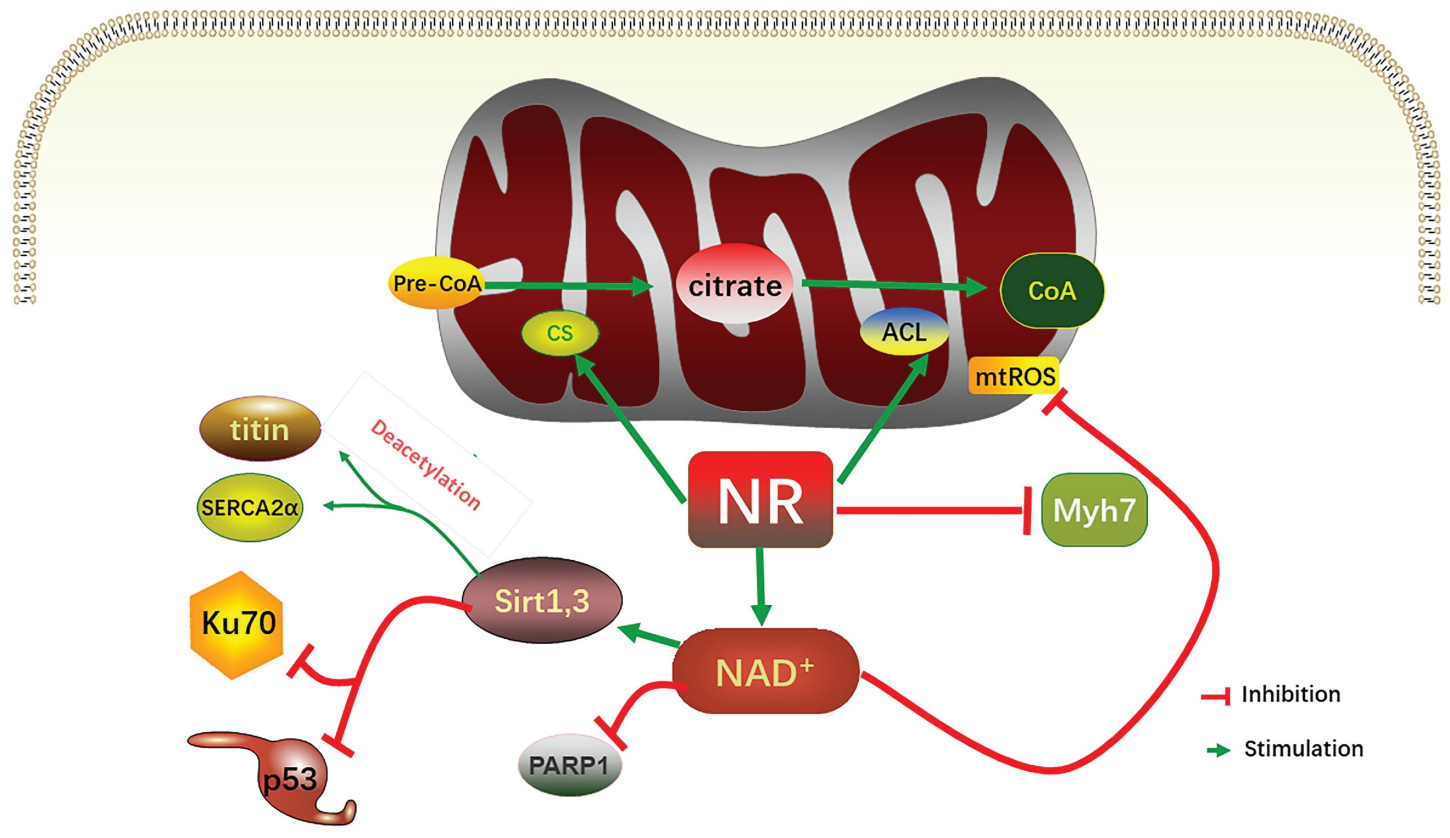
Relevant mechanisms of increasing NAD^+^ levels alleviating heart failure. In mitochondria, NR increased the activities of CS and ACL, increased CoA, and acetylated nucleoprotein. NR limited induction levels of Myh7; NR increases the NAD^+^ level to inhibit the activity of PARP1, and increases the activity of sirt1 and sirt3, which in turn inhibits P53 and Ku70, to deacetylate titin and SERCA2α, two relaxation regulatory proteins. The increase in NAD^+^ level also decreases mitochondrial ROS. NR, nicotinamide ribonucleoside; CS, mitochondrial citrate synthase; ACL, TP- citrate lyase; CoA, acetyl-coA, mtROS, mitochondrial reactive oxygen species; PARP, poly (ADP- ribose) polymerase.
